# Glutathione and Jasmonic Acid Biosynthesis Coordinate Antioxidant and Hormonal Responses to Alleviate Lead Toxicity in *Pogonatherum crinitum* Roots

**DOI:** 10.3390/plants15152288

**Published:** 2026-07-26

**Authors:** Weicai Meng, Leilin Qiu, Yueli Du, Yuqi Yuan, Yijie Li, Xiaoyu Wang, Yang Hu, Xiaolong Hou

**Affiliations:** 1 College of Forestry, Fujian Agriculture and Forestry University, Fuzhou 350002, China; 2 Gansu Bailongjiang Forestry Protection Center, Lanzhou 730050, China; 3 Key Laboratory of Soil and Water Conservation of Southern Red Soil Region, State Forestry and Grassland Administration, Fuzhou 350002, China; 4 National Positioning Observation and Research Station of Red Soil Hilly Ecosystem, Changting, Longyan 364000, China; 5 Co-Innovation Center for Soil and Water Conservation in Red Soil Region of the Cross-Strait, Fuzhou 350002, China

**Keywords:** *Pogonatherum crinitum* (Thunb.) Kunth, metabolomics, proteomics, integrative omics analysis, Pb stress

## Abstract

Multiomics is increasingly valued as a strategy for investigating the regulatory mechanisms by which plants respond to adverse stress conditions. Currently, information on the molecular processes underlying plant responses to Pb stress, particularly those observed through an approach that combines proteomics and metabolomics, is lacking. Therefore, in this study, we aimed to explore functional correlations between Pb-responsive proteins and metabolites under Pb stress by performing label-free quantitative proteomics and untargeted metabolomics on the roots of the Pb hyperaccumulator *Pogonatherum crinitum* (Thunb.) Kunth. The selected Pb stress-responsive proteins were functionally verified using quantitative reverse transcription polymerase chain reaction (RT-qPCR) and parallel reaction monitoring (PRM). Under Pb stress, 397 upregulated and 431 downregulated proteins were identified through proteomic analysis. Metabolomic analysis identified 478 upregulated and 354 downregulated metabolites. Pathway enrichment analysis using the Kyoto Encyclopedia of Genes and Genomes revealed that differentially expressed proteins and metabolites were involved in pathways linked to heavy metal stress, such as starch and sucrose metabolism and plant hormone signal transduction. Through integrated proteomic and metabolomics analyses, we uncovered the coordinated regulatory interplay between glutathione (GSH) metabolism and jasmonic acid signaling. GSH reductase and 12-oxophytodienoate reductase drive the accumulation of GSH and jasmonic acid, respectively. The synergistic enhancement of these components is critical for maintaining cellular redox homeostasis and activating hormone-mediated defense signaling. These downstream metabolites were upregulated under Pb stress. RT-qPCR validation revealed that the transcriptional change trends were consistent with those of the proteomics analysis. Further quantitative validation of the target protein using PRM revealed significant upregulation under Pb stress. In conclusion, the *P. crinitum* root system upregulated the activity of key enzymes in the antioxidant system and plant hormone synthesis under Pb stress, thereby regulating the accumulation of GSH, glutamate, and metabolites for jasmonic acid synthesis. This integrated regulatory network provides promising candidate targets for breeding Pb-tolerant hyperaccumulators to remediate Pb-contaminated farmland and mining soil.

## 1. Introduction

Heavy metal pollution of soil and soil remediation are universally concerning environmental issues. Approximately 14–17% of the world’s cultivated land (~242 million hectares) has been contaminated by toxic heavy metals, affecting an estimated 0.9–1.4 billion people [1]. In China, Pb exceeds the standard level at 1.5% of surveyed sites and is listed as one of the key pollutants in the National Soil Pollution Survey Bulletin, posing a particularly acute threat to public health and safety [2]. Against this backdrop, the challenges of heavy metal-contaminated soils and their remediation have drawn worldwide attention. In plants, Pb is a non-essential trace metal whose accumulation in plant tissues can affect normal growth and development [3,4,5]. Moreover, Pb accumulation in the environment can affect animals and humans, causing harm to multiple physiological systems, including the nervous, digestive, hematopoietic, and renal systems, making Pb pollution of soil a public health issue [6,7]. Phytoremediation technology is favored for mitigating Pb contamination of soil because of its low-carbon and environmentally friendly nature. However, the disadvantages of this approach are low biomass and low remediation efficiency [8,9]. Therefore, understanding the mechanisms by which plants can withstand Pb can help to improve phytoremediation technologies.

Under Pb stress, the aboveground Pb concentration in the hyperaccumulator *Sedum alfredii* Hance reaches 152.8–460 mg·kg^−1^, with a bioconcentration factor of 0.5–1.2 [10,11]. *Lolium perenne* L. exhibits a bioconcentration factor of 0.12–0.88 [12] and a translocation factor of 0.11–0.42 [13]. Although several plant species accumulate Pb, their bioconcentration and translocation factors are generally low. In contrast, *Pogonatherum crinitum* (Thunb.) Kunth is a perennial herbaceous plant with strong Pb tolerance and accumulation capacity. This species can grow normally in soils containing up to 17,496 mg·kg^−1^ Pb, with a bioconcentration factor as high as 10.18 and a translocation factor of 1.14–1.32, indicating its strong potential for Pb accumulation and translocation [14]. Therefore, *P. crinitum* exhibits high tolerance to Pb-contaminated soils and its bioconcentration and translocation capacities are markedly superior to those of other species.

The level of Pb in *P. crinitum* is lowered to less than toxic levels through increased root biomass allocation, rapid root elongation, and accelerated tiller renewal [15]. Physiological changes include enhancement of antioxidant capacity in roots by regulating catalase activity, the AsA-GSH cycle, and osmotic regulatory substances [16]. At the transcriptional level, CL14444.Contig1 in this species is upregulated, prompting the synthesis of downstream substances such as lignin, which increases cell wall strength and thus confers tolerance to Pb toxicity. Pb stress induces the upregulation of GSH reductase (CL12071.Contig1) and glutathione peroxidase (GPX) (CL13989.Contig3) in the roots of *P. crinitum*, thereby promoting the production of glutathione (GSH) when oxidized glutathione (GSSG) is reduced, which maintains antioxidant capacity and enhances stress resistance [17].

Previous studies have provided a foundation for understanding the mechanisms underlying Pb tolerance in *P. crinitum*. However, research has primarily focused on morphological, physiological, and transcriptomic responses. Studies investigating the responses of *P. crinitum* roots to Pb stress from proteomic and metabolomic perspectives remain limited. Consequently, the underlying protein–metabolite regulatory mechanisms remain unclear.

Several studies have examined how plants react to heavy metal stress at the molecular level [18,19,20,21]. Proteomic and metabolomic analyses have been used to elucidate how plants react to heavy metal stress, including Pb stress [22,23]. The reported evidence suggests that plants can alleviate toxicity through heavy metal chelation and antioxidant systems and by regulating molecular signaling networks, thus modulating metabolic pathways [19,24,25]. Al and Pb stress can induce significant upregulation of proteins related to phenylpropanoid biosynthesis and metal transport (phenylalanine ammonia-lyase, cinnamyl alcohol dehydrogenase, and peroxidase) in the roots of *Triticum aestivum* L. [26] and *Miscanthus lutarioriparius* L. [27], and these proteins could be part of the reaction process of the roots to reach high levels of heavy metals.

Metabolomics can provide insights into the metabolic regulation of heavy metals [22,28]. Metabolomic analysis has revealed that under Pb stress, *Cuminum cyminum* L. may reduce Pb toxicity in plants by enhancing the content of three types of metabolic products in the roots (amino acids, organic acids, and carbohydrates) [29]. In a low-Pb environment (Pb500), the content of metabolic products, such as ornithine, citrate, and isocitrate, is significantly increased in *Trifolium pratense* L., and these metabolic products play a role in Pb stress [30]. However, data generated from a single omics approach are insufficient to fully explain the complexity of biological processes. To overcome these limitations, proteomics and metabolomics can be integrated for metabolic pathway enrichment analysis to reveal molecular processes and stress response mechanisms [31,32].

Kuang et al. combined proteomic and metabolomic analyses to show that the phenylpropanoid and tryptophan metabolism pathways were significantly enriched in the root tips of *Oryza sativa* L. under Cd stress [33]. Under Pb stress, *C. cyminum* alleviates Pb toxicity by strengthening the metabolic pathways for GSH and plant hormone signaling [29]. An integrated proteomic and metabolomic study of *Populus × canescens* (Aiton) Sm. under Pb stress showed that plants could maintain metabolic and phenotypic stability, even in the absence of visible phenotypic damage, by enhancing proteomic responses associated with Pb immobilization, cytoskeletal remodeling, H+-ATPase regulation, and stabilization of protein turnover [34]. These findings demonstrate that integrating proteomics and metabolomics into a multiomics analysis reveals adaptive regulatory processes difficult to capture alone. Ultimately, this approach provides a clearer view of the complex response mechanisms plants use to survive heavy metal stress.

This study is the first to use a combination of label-free proteomic quantitative technology and untargeted metabolomics technology to systematically identify differentially expressed metabolites (DEMs) and proteins (DEPs) in the Pb-hyperaccumulating grass *P. crinitum* under Pb stress. This approach deepens our understanding of the molecular mechanisms by which *P. crinitum* responds to Pb stress.

We utilized the Kyoto Encyclopedia of Genes and Genomes (KEGG) database for the annotation of metabolic pathways and functional analysis, examined the variations in metabolites downstream of proteins involved in Pb stress response in *P. crinitum* roots, and verified the authenticity and accuracy of the proteomics data using reverse transcription quantitative polymerase chain reaction (RT-qPCR) and parallel reaction monitoring (PRM) technology with high-resolution mass spectrometry. The objective of the present study was to elucidate the molecular mechanisms underlying the response of *P. crinitum* to Pb stress by integrating proteomics and metabolomics. To achieve this. We mapped the two omics layers onto common metabolic pathways and identified the relationships between significantly upregulated proteins and their downstream metabolites to uncover how *P. crinitum* responds to Pb stress at the molecular level. The findings of this study provide a basis for elucidating the molecular mechanisms underlying plant response to Pb stress.

## 2. Results

### 2.1. Protein and Metabolomic Analysis

**Figure 1:**
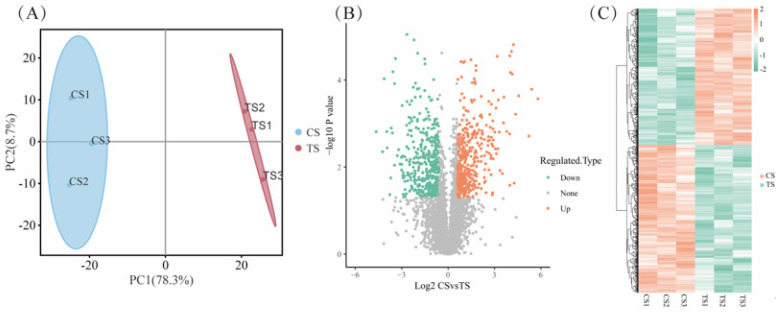
Principal component analysis of proteomics data from the root systems of *Pogonatherum crinitum* (**A**). Volcano plot of proteins from the root systems of *P. crinitum* (**B**). Cluster heatmap of DEPs in the root systems of *P. crinitum* (**C**). Note: CS represents the Pb-free control group (CK), and TS represents the treatment group subjected to 1000 mg·L^−1^ Pb stress (Pb1000).

**Figure 2:**
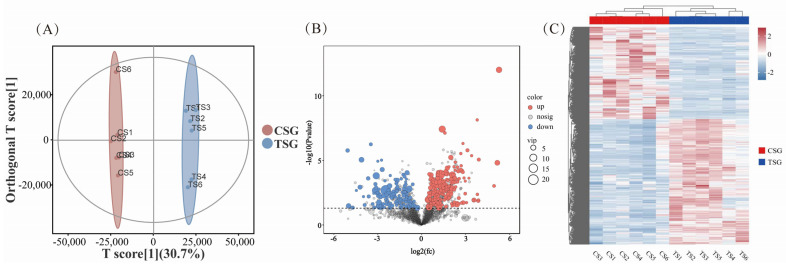
Orthogonal partial least squares discriminant analysis (OPLS-DA) score plot of metabolites in the root systems of *P. crinitum* (**A**). Volcano plot of metabolites from the root systems of *P. crinitum* (**B**). Cluster heatmap of DEMs in the root systems of *P. crinitum* (**C**). Note: CSG represents the Pb-free control group (CK), and TSG represents the treatment group subjected to 1000 mg·L^−1^ Pb stress (Pb1000).

In total, 213,749 valid spectra were detected in *P. crinitum* roots, identifying 35,012 peptides and 7773 proteins. Among these, 5328 proteins (68.5% of total identified proteins) were quantified using unique peptides (Supplementary Table S2). The Pb stress treatment and control groups differed significantly in protein aggregation locations in the *P. crinitum* root systems (Figure 1A). A total of 828 proteins were differentially expressed in the *P. crinitum* root system under Pb stress, with 397 proteins upregulated (47.9%) and 431 downregulated (52.1%) (Figure 1B,C). In accordance with the orthogonal partial least squares discriminant analysis (OPLS-DA) (variable importance in projection [VIP] > 1, *p* < 0.05, Figure 2A), no overlapping areas were found between the treatment and control groups, indicating differences in the metabolites between the two treatments. A total of 832 distinct metabolites were detected, of which 478 were upregulated and 354 were downregulated under Pb stress (Figure 2B,C).

### 2.2. KEGG Enrichment Analysis of Proteins and Metabolites

**Figure 3:**
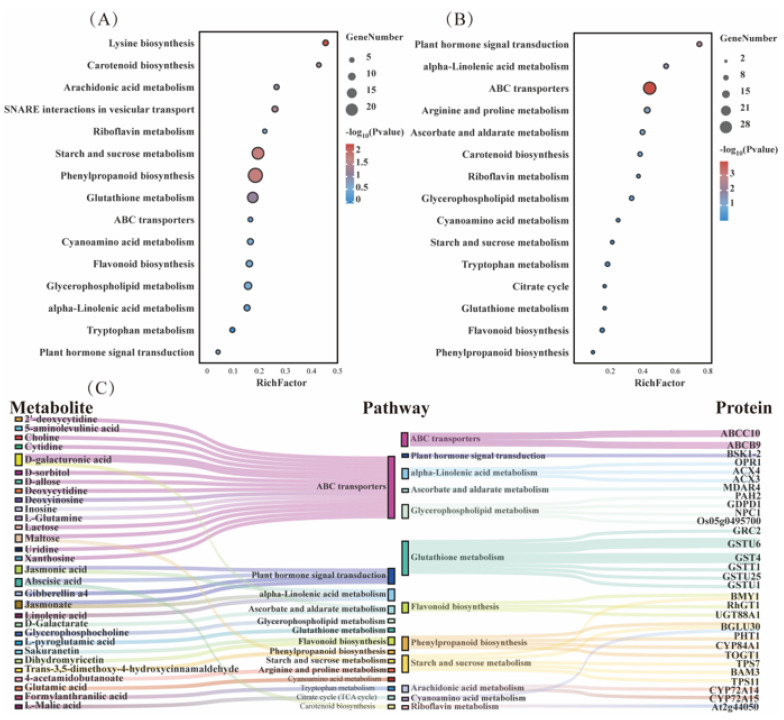
Kyoto Encyclopedia of Genes and Genomes (KEGG) enrichment analysis of DEPs (**A**) and differential metabolites (**B**) in *P. crinitum* root systems. Sankey diagram illustrating metabolic pathway involvement of proteins and metabolites involved in Pb stress response in *P. crinitum* root systems (**C**).

The DEPs and DEMs in the *P. crinitum* root system were functionally annotated and subjected to pathway enrichment analysis using the KEGG database. As shown in Figure 3A,B, 116 DEPs were annotated in 15 metabolic pathways related to Pb tolerance, and 90 DEMs were enriched in 15 metabolic pathways associated with Pb tolerance.

The proteomic and metabolomic analyses indicated that the shared metabolic pathways related to Pb exposure included ABC transporters, plant hormone signal transduction, carotenoid biosynthesis, and starch and sucrose metabolism. The carotenoid biosynthesis, starch and sucrose metabolism, and phenylpropanoid biosynthesis pathways were significantly enriched in the proteomics analysis (*p* < 0.05; Figure 3A). In the metabolomics analysis, ABC transporters, the alpha-linolenic acid metabolism pathway, and plant hormone signal transduction were significantly enriched (*p* < 0.05; Figure 3B).

As shown in Figure 3C, after analyzing the 15 metabolic pathways related to Pb, we identified 30 upregulated DEPs and DEMs (*p* < 0.05), including components of the metabolic pathways involved in the antioxidant enzyme system, ascorbate and aldarate metabolism, riboflavin metabolism, GSH metabolism, flavonoid biosynthesis, and phenylpropanoid biosynthesis. Both DEPs and DEMs in the *P. crinitum* root system under Pb stress were significantly enriched in metabolic pathways related to these antioxidant enzymes, accounting for 20% and 50%, respectively. The DEPs β-amylase (PcBMY1) and β-glucosidase (PcBGLU30) and the DEM maltose were found to be involved in the starch and sucrose metabolism pathway. In the plant hormone signal transduction pathway, an enzyme, a serine/threonine protein kinase (PcBSK1-2), and a metabolite (jasmonic acid) were upregulated.

### 2.3. Metabolic Pathway Analysis of DEPs and DEMs

**Figure 4:**
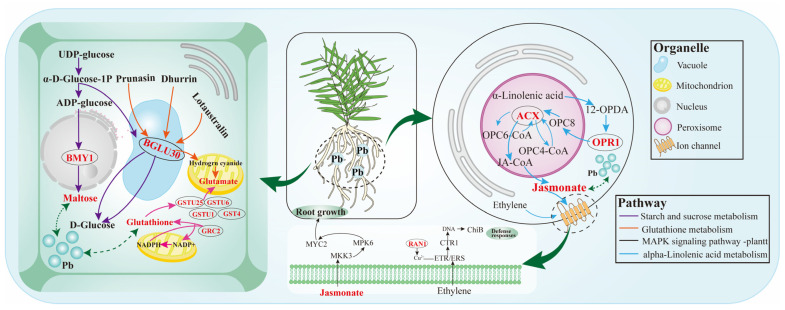
Metabolic pathways involving the DEPs and metabolites in the root systems of *P. crinitum* under Pb stress.

To further understand the biological significance of Pb stress-responsive proteins and metabolites in *P. crinitum* under Pb stress, the metabolic processes involving these proteins and metabolites were examined using the KEGG pathway database (Figure 4). In the starch and sucrose metabolic pathway, β-amylase (PcBMY1) functions in the nucleus, with its upstream product being UDP-glucose, which is ultimately converted into maltose through upregulated expression. β-glucosidase (PcBGLU30) functions in the vacuole and ultimately forms D-glucose through upregulated expression. These soluble sugars act as solutes in the regulation of osmotic pressure within plant cells. This indicates that when *P. crinitum* is subjected to Pb stress, an increase in the soluble sugar content can reduce its osmotic potential, thereby resisting damage resulting from Pb stress. In the GSH metabolic pathway, glutathione reductase (GR) (PcGRC2) and glutathione S-transferase (GST) (PcGSTU25, PcGSTU6, PcGSTU1, and PcGST4) function in the cytoplasm. In the GSH redox cycle, PcGRC2 reduces GSH, with NADPH acting as an electron donor. Thus, these cells are protected from oxidative damage. The upregulated expression of GST (PcGSTU25, PcGSTU6, PcGSTU1, and PcGST4) results in the production of glutamic acid because the enzymes acting on GSH act as reaction substrates. In alpha-linolenic acid metabolism, the upregulation of 12-oxophytodienoate reductase (PcOPR1) and acyl-coenzyme A oxidase (PcACX4) results in the synthesis of jasmonic acid, which acts as a signaling molecule in the MAPK signaling pathway, resulting in the detoxification of Pb during *P. crinitum* root development.

### 2.4. Protein Metabolic Interaction Network Analysis

**Figure 5:**
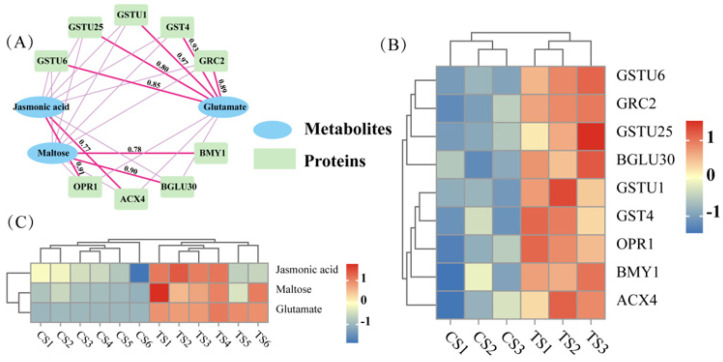
Changes in the *P. crinitum* root system induced by Pb stress. Metabolic interaction network diagram of proteins involved in Pb stress response (**A**), protein heatmap from proteomics analysis (**B**), and metabolite heatmap from metabolomics analysis (**C**). Note: In (**A**), edges represent Pearson correlation coefficients indicating the correlation strength between the expression patterns of connected molecules. In (**B**) and (**C**), the Pearson correlation heatmaps show the pairwise correlation of samples between groups.

We performed an interaction network analysis of the selected Pb stress-responsive proteins and metabolites (Figure 5A) and found that the levels of GST (PcGSTU25, PcGSTU6, PcGSTU1, and PcGST4) and GR (PcGRC2) were significantly and positively correlated with the levels of glutamic acid, with correlation values of 0.80, 0.85, 0.97, 0.93, and 0.89, respectively. These findings suggest that these enzymes participate in the regulation of glutamic acid synthesis and antioxidant defenses in *P. crinitum* root systems. PcBMY1 expression showed a significant positive correlation with maltose levels. Given that PcBMY1 is related to carbohydrate metabolism, its activation of PcBMY1 in *P. crinitum* likely accelerates sugar metabolism. The levels of both PcOPR1 and PcACX4 were significantly positively correlated with those of the metabolite jasmonic acid (correlation coefficient 0.91), indicating that changing the levels of plant hormones enhanced the activity of the antioxidant enzyme system in *P. crinitum* under Pb stress.

By integrating the results from the proteomic and metabolomic analyses (Figure 5B,C), we found that Pb stress-responsive proteins and metabolites were upregulated under Pb stress. This further confirmed that *P. crinitum* can increase its resistance to Pb stress by increasing the content of these proteins and metabolites.

### 2.5. Verification of Pb-Stress-Responsive Proteins and Metabolites

**Figure 6:**
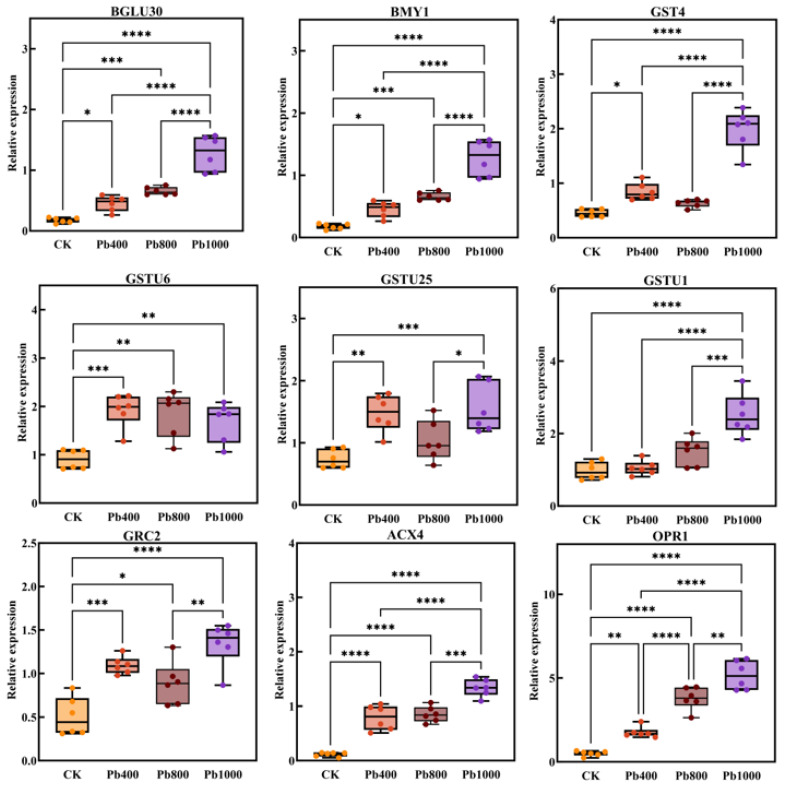
Expression of DEPs under various Pb treatment concentrations. Note: * indicates significant difference (*p* < 0.05), ** indicates significant difference (*p* < 0.01), *** indicates significant difference (*p* < 0.001), and **** indicates significant difference (*p* < 0.0001).

The results obtained for proteins and metabolites involved in Pb stress response in the *P. crinitum* root system were verified at the transcriptomic level using RT-qPCR. As shown in Figure 6, the relative expression levels of the five antioxidant-related genes encoding DEPs increased as Pb concentration increased. The relative expression levels of *PcGSTU25* and *PcGRC2* were significantly higher in the Pb treatment groups than in the CK (*p* < 0.05) and peaked under the Pb1000 treatment, increasing by 2.10 and 2.65 times compared to the corresponding levels in the CK. The proportional degrees of expression of *PcOPR1* and *PcACX4*, which are involved in the biosynthesis of jasmonic acid, were markedly higher in the Pb400, Pb800, and Pb1000 treatment groups than in the CK (*p* < 0.05) and increased with increasing Pb treatment concentration. The proportional degrees of expression of *PcBMY1* and *PcBGLU30*, which are involved in carbohydrate metabolism, increased with increasing Pb treatment concentration and were significantly higher in the Pb800 and Pb1000 treatment groups than in the CK (*p* < 0.05), being 3.82- and 7.22-times higher, respectively.

The levels of nine proteins involved in Pb stress response were elevated at both the transcriptome and proteome levels. These proteins were selected for quantitative analysis using PRM to verify the label-free proteomic results (Figure 5A). Preliminary experiments revealed that six proteins (PcBGLU30, PcBMY1, PcGST4, PcGSTU1, PcGRC2, and PcACX4) could be detected using PRM (Supplementary Table S3). PRM validation revealed that these six Pb-resistant proteins were upregulated following the Pb400, Pb800, and Pb1000 treatments. We also found that the expression levels of PcBGLU30, PcBMY1, and PcGRC2 increased as the Pb concentration increased. This result was consistent with the results of proteomic analysis (Supplementary Table S4) and transcriptome levels determined using RT-qPCR.

**Figure 7:**
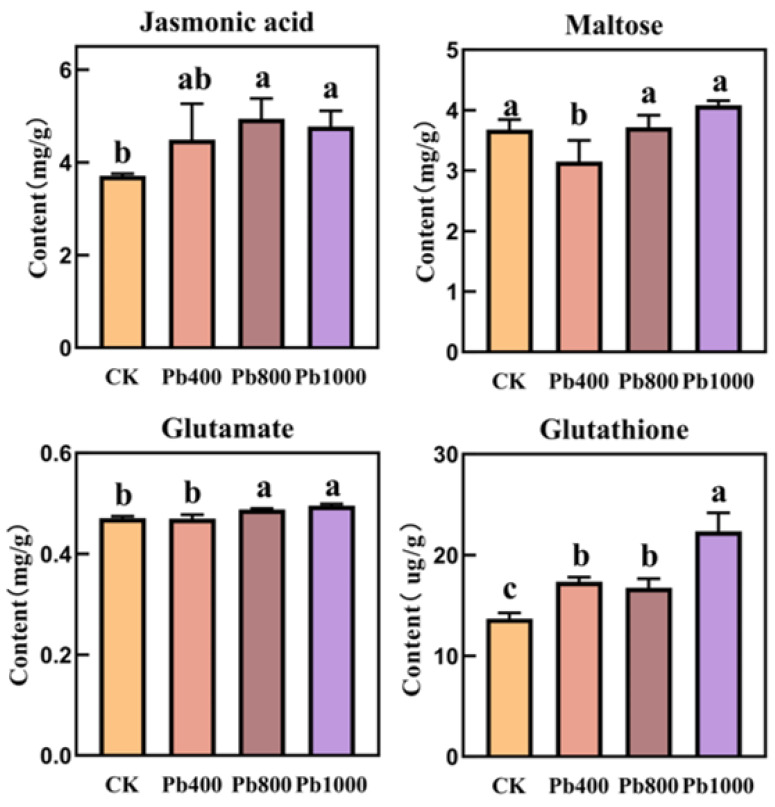
Levels of downstream metabolites of proteins involved in Pb stress response under varying concentrations of Pb. Different lowercase letters indicate significant differences (*p* < 0.05) for the same indicator between treatments according to Duncan’s multiple range test.

Further analysis of the protein metabolic pathways with variable expression revealed four key downstream metabolites that may be significant factors in the adaptability of *P. crinitum* roots to Pb stress (Figure 7). The jasmonic acid content, regulated by PcACX4, indicated a rising trend followed by a fall when Pb treatment concentrations increased, peaking under the Pb800 treatment at 4.94 mg·g^−1^, which was 33.1% higher than that of the CK. The GSH content, regulated by PcGRC2, was highest under the Pb1000 treatment, reaching 22.35 µg·g^−1^. This was significantly greater than that of the other groups (*p* < 0.05), being 1.63, 1.28, and 1.33 times the levels of the CK, Pb400, and Pb800 groups, respectively. Glutamic acid, the downstream target of PcGSTU1 and PcGST4, was not significantly different between treatment groups. The content of maltose, the downstream substance regulated by PcBMY1, showed a decreasing and then increasing trend with increasing Pb treatment concentration and the highest, at 4.08 mg/g, following Pb1000 treatment.

## 3. Discussion

### 3.1. PcGSTs/PcGRC2-Mediated Glutathione Cycling Enhances Pb Detoxification in P. Crinitum

Under heavy metal Pb stress, *P. crinitum* can strengthen its detoxification system by enhancing antioxidant capacity. In this study, the upregulated Pb-responsive proteins in the roots of *P. crinitum*, including GST (PcGSTU25, PcGSTU6, PcGSTU1, and PcGST4) and GR (PcGRC2), were associated with the GSH metabolic pathway. This coordinated detoxification and antioxidative strategy, centered on GSH metabolism, is a key component of plant responses to heavy metal stress. This finding is consistent with the results of Yang et al. [29]. For *C. cyminum*, most genes encoding GSH-related metabolic enzymes were upregulated, thereby promoting GSH synthesis and alleviating Pb-induced cellular damage. Similarly, under Cd stress, genes involved in GSH metabolism are significantly upregulated in *Morus alba*, *Brassica napus*, and *Medicago sativa*, promoting the production of GSH and phytochelatins (PCs) and enhancing heavy metal tolerance through root cell wall compartmentalization [35,36,37]. This is because GSH, as the most abundant non-protein thiol in cells [38,39], plays a key role in the detoxification of Pb by virtue of its high-affinity thiol groups. Integrated proteomic and metabolomic analysis revealed that PcGRC2 contributed to GSH regeneration through the redox cycle, which is consistent with previous transcriptomic evidence showing that GSH reductase was upregulated in the roots of *P. crinitum* under Pb treatment and promoted the conversion of GSSG to GSH [17].

Riboflavin metabolism plays a key role in the proteome of *P. crinitum* under Pb stress. Under stress conditions, the remodeling of riboflavin metabolism helps maintain redox homeostasis and supports respiratory metabolism and can also function as an elicitor of defense responses [40]. Riboflavin acts as an antioxidant during heavy metal stress by improving redox balance, thereby reducing the toxic effects of heavy metals on plants and protecting cells from oxidative damage [41]. The enhancement of carotenoid metabolism can improve the antioxidant capacity of plants, thereby alleviating the toxic effects of heavy metals. Therefore, the response mechanism of *P. crinitum* to Pb stress may involve modulating riboflavin, carotenoid, and GSH-related metabolic pathways to strengthen its antioxidant capacity. Khan et al. reported that exogenous reduced GSH alleviated Pb-induced Reactive Oxygen Species (ROS) damage in *Gossypium hirsutum* L. and helped maintain the stability of subcellular structures in roots and leaves [42]. The present study further revealed that Pb stress induced coordinated responses of riboflavin-, carotenoid-, and GSH-related pathways in *P. crinitum*, suggesting that *P. crinitum* may not rely on a single antioxidant mechanism under Pb stress, but instead maintains cellular stability through the integrated regulation of redox homeostasis across multiple pathways.

### 3.2. PcBMY1-Mediated Maltose Metabolism Enhances Pb Detoxification in P. Crinitum

Our study revealed that starch and sucrose metabolic pathways are involved in the response of *P. crinitum* to Pb stress and ensure energy supply. Specifically, the relative expression level of β-amylase (PcBMY1) in the roots of *P. crinitum* increased with increasing Pb concentrations. Under heavy metal stress, heat shock proteins in plant cells promote the correct folding of key metabolic enzymes including β-amylase, thereby improving plant adaptation to adverse conditions [43,44]. This suggests that heat shock proteins in *P. crinitum* roots may interact with β-amylase under Pb stress, thereby affecting energy metabolism and stress responses. Integrated proteomic and metabolomic analysis further showed that PcBMY1 upregulation was associated with maltose accumulation in *P. crinitum* roots. In Pb-resistant genotypes of *M. sativa*, β-amylase abundance increases with increasing Pb stress and may contribute to higher soluble sugar content [45]. Wei et al. [46] reported that soluble sugars function not only as energy reserves but also as osmotic regulators and signaling molecules that play essential roles in plant growth, development, and stress responses. Therefore, the increased soluble sugar content observed in the roots of *P. crinitum* likely represents an adaptive response to Pb stress. These results indicate that *P. crinitum* roots alleviate Pb toxicity by enhancing soluble sugar accumulation, particularly maltose.

### 3.3. PcOPR1/PcACX4-Mediated Phytohormone Signaling Enhances Pb Detoxification in P. Crinitum

Under heavy metal stress, excessive ROS accumulation can activate GSH metabolism and antioxidant enzyme systems, while ROS and redox molecules such as GSH/GSSG can also interact with phytohormone signaling to collectively regulate plant detoxification and stress adaptation processes [47]. Phytohormones such as abscisic, jasmonic, and salicylic acids can enhance plant tolerance to heavy metal stress by modulating GSH accumulation, phytochelatin synthesis, antioxidant enzyme activity, and metal transport-related processes [48,49,50]. Therefore, the interplay between antioxidant metabolism and phytohormone signaling represents a regulatory network that coordinates detoxification, oxidative damage mitigation, and growth adaptation in plants under heavy metal stress.

Under Pb stress, *P. crinitum* roots regulate hormone biosynthesis and signaling through the plant hormone signal pathway, thereby mitigating Pb toxicity. Li et al. [51] found that under Pb stress, the gene Os01g0382000, which encodes PR1, participates in salicylic acid signaling during the stress response. Salicylic acid promotes plant growth and enhances tolerance to heavy metal stress [52], partly by stimulating the synthesis of GSH and phytochelatins, thereby improving cellular survival under metal stress [53]. Our study found that PcOPR1 and PcACX4 were both upregulated in the α-linolenic acid metabolism pathway under Pb stress.

Under Cd stress, the Cd-tolerance-related gene OsOPR1 is induced in the roots of *O. sativa*, and OsOPR1 overexpression reduces Cd accumulation in roots while affecting the expression of antioxidant enzyme-related genes, thereby enhancing Cd tolerance [54]. Subcellular localization studies have shown that OPR1 in *Nicotiana tabacum* L., OPR7 in *O. sativa*, and OPR3 in *Solanum lycopersicum* Mill. are localized to peroxisomes and participate in plant heavy metal stress response [55,56]. Therefore, OPR1 is highly expressed in plant roots and is primarily involved in plant defense [57]. These findings are consistent with our observations, indicating that under Pb stress, PcOPR1 in *P. crinitum* functions in peroxisomes.

Protein–metabolite interaction network analysis revealed that PcOPR1 and PcACX4 were significantly and positively correlated with jasmonic acid. Beynon et al. [58] and Aoki et al. [59] found that OPR1 is involved in jasmonic acid biosynthesis and defense-related processes in *Arabidopsis thaliana* (L.) Heynh. and *S. lycopersicum*. Jasmonic acid and gibberellin are phytohormones that regulate plant defense responses and growth and development, respectively [60]. These hormones can alleviate heavy metal stress mainly by promoting plant growth, increasing antioxidant capacity, and reducing heavy metal uptake and transport [61]. In *S. lycopersicum*, jasmonic acid treatment can reduce Pb uptake and enhance the AsA-GSH cycle [62]. In *A. thaliana*, jasmonic acid can alleviate Cd toxicity by inhibiting Cd uptake and long-distance translocation [63]. In our study, jasmonic acid appeared to function as a signaling molecule involved in the regulation of root growth in *P. crinitum* under Pb stress. This interpretation is consistent with the findings of Liu [64] and Li et al. [65], who reported that exogenous jasmonic acid and gibberellin alleviate the inhibitory effects of Cd stress and salt stress on root growth in *O. sativa* and *Sorghum bicolor (L.) Moench*, by regulating the antioxidant defense system and promoting chelation. Chen et al. [66] reported that jasmonic acid and its derivatives participate in plant tolerance to heavy metals and metalloids by coordinating ion transport systems, antioxidant enzyme activity, and chelating capacity. However, the underlying molecular mechanisms have only been validated in a few species, such as *A. thaliana* and *O. sativa*. In the present study, we elucidated, at the molecular level, that PcOPR1 is upregulated under Pb stress in *P. crinitum*, thereby activating the jasmonic acid signaling pathway and alleviating Pb toxicity. Collectively, these results indicate that under Pb stress, the roots of *P. crinitum* may alleviate Pb toxicity by enhancing jasmonic acid-associated signaling, thereby influencing root growth and activating antioxidant defense pathways.

## 4. Conclusions

The tolerance mechanism of *P. crinitum* under Pb stress does not rely on a single oxidative stress response but rather on the coordinated response of multiple pathways, including the redox regulation of GSH, riboflavin, and carotenoids, sugar metabolism, and jasmonic acid signal transduction. This synergistic regulatory strategy enables *P. crinitum* to maintain root function under Pb stress. The major finding of this study is that upregulated PcGRC2 promotes GSH synthesis, thereby activating the antioxidant enzyme system. Upregulated PcOPR1 activates jasmonic acid signal transduction. These findings reveal the molecular mechanism by which *P. crinitum* roots respond to Pb stress through the coordinated synthesis of GSH and jasmonic acid, integrating redox homeostasis, hormone signaling, and energy metabolism. This provides a theoretical basis for a deeper understanding of the regulatory network of plant heavy metal detoxification and for the application of *P. crinitum* in the phytoremediation of Pb-contaminated soils.

## 5. Materials and Methods

### 5.1. Experimental Materials

The materials used in the experiment were new *P. crinitum* seedlings that sprouted from tillers in the Pb-Zn mining area of Sanming City, Fujian Province, China. The specimen of *P. crinitum* is available in the Flora of China (https://www.iplant.cn/foc) (accessed on 27 May 2025) and is deposited in the Digital Herbarium of China (https://www.cvh.ac.cn/) (accessed on 27 May 2025) under the accession number PE 01554012. Seeds were evenly sown in nutrient soil and cultivated for approximately 60 days under greenhouse conditions. Once the seedlings entered the vegetative growth stage, healthy plants with uniform growth, approximately 13 cm in height, and free from pests and diseases were selected for stress treatments. A lead acetate (analytical grade) stock solution with a Pb concentration of 60 g·L^−1^ was prepared for proteomic, metabolomic, RNA extraction, RT-qPCR, PRM, and downstream metabolite determination experiments.

### 5.2. Experimental Design

Prior to transplantation, the roots were rinsed with tap water and washed three times with deionized water to remove adhering soil particles and impurities. The cleaned plants were transplanted into 1/8 Hoagland nutrient solution [67] at a density of four plants per container for 3 days to acclimatize to the hydroponic conditions and restore growth vigor. Uniform *P. crinitum* seedlings with consistent growth status and no significant differences in plant height and root length were selected for stress treatments. Two sets of stress treatment experiments were established: (1) Proteomics and metabolomics analysis experiments: Hydroponic treatments included a Pb-free control group (CK) and a 1000 mg·L^−1^ Pb treatment group (Pb1000) (determined according to the literature [17]. Each treatment consisted of three biological replicates. Each biological sample was subjected to two technical replicates to assess instrument stability [68,69,70]. (2) Validation experiment: Based on omics assays and preliminary experiments, we established hydroponic treatments consisting of a Pb-free control (CK) and three Pb-stress concentrations, 400 mg·L^−1^ (Pb400), 800 mg·L^−1^ (Pb800), and 1000 mg·L^−1^ (Pb1000), to investigate the dynamic response of plants to different Pb stresses [17]. Each treatment comprised three biological replicates, and each biological sample had two technical replicates. The plants were placed in a climate-controlled chamber (temperature: 22 °C; humidity: 75%; light intensity: 81 μmol·m^−2^ s^−1^; photoperiod: 16 h light/8 h dark) for stress treatment. To promote growth, the hydroponic system was aerated twice daily (morning and evening) for 15 min in each session. After aeration, the 1/8 Hoagland nutrient solution was replenished to maintain the initial stress solution level. Following three days of stress treatment (determined based on previous hydroponic studies [71,72,73] and preliminary trials to detect protein and metabolite stress responses), *P. crinitum* plants were harvested. Roots were washed with deionized water, blotted dry with filter paper, and fresh young root tips (6 cm segments with active cellular activity) were collected. The samples were immediately frozen in liquid nitrogen and stored at −80 °C for analyses.

### 5.3. Measurement Methods

#### 5.3.1. Proteomic Sequencing Methods

The proteomic analysis was performed using a label-free quantitative analytical method. A 200-mg sample of *P. crinitum* root was placed in a mortar, liquid nitrogen was added, and the sample was thoroughly ground into a powder. Four times the amount of 10% trichloroacetic acid/acetone was added, and the sample was allowed to stand before being centrifuged. Acetone was used to wash the precipitate, and the supernatant was air-dried for sodium dodecyl sulfate-phenol extraction. To determine protein concentration, a bicinchoninic acid assay kit was used. Protein samples were subjected to enzymatic digestion in a lysis buffer. First, dithiothreitol and iodoacetamide were added for alkylation, after which the solution was replaced with urea. A buffer was used to replace the solution, and trypsin was added for digestion. After digestion, peptides were recovered by centrifugation. The peptide samples were loaded onto an EASY-nLC 1200 ultra-high-performance liquid chromatography system and separated using mobile phase A. After ionization via injection into the NSI ion source, the separated peptides were examined using an Orbitrap Explorer 480 mass spectrometer. Raw mass spectrometry data were quality controlled using QuiC 5 software (Biognosys). Pulsar X v17 software was used for database construction, and DESeq2 [74] was used for differential protein expression analysis. DEPs were screened using the following criteria: P (Q) < 0.05 and fold change (FC) > 1.5 indicated upregulated proteins, and FC < 0.667 (or equivalently, FC < 1/1.5) indicated downregulated proteins. The identified proteins were subjected to functional enrichment analysis using the Gene Ontology (GO) and KEGG databases.

#### 5.3.2. Metabolomics Sequencing Methods

*P. crinitum* root samples (250 mg) were added to a pre-cooled mixture of water, acetonitrile, and methanol (1:2:2 volume ratio) and thoroughly mixed using a vortex. After treatment under low-temperature ultrasonic conditions for 30 min, the samples were allowed to stand at −20 °C for 10 min, after which the material was centrifuged at 14,000× *g* at 4 °C for 20 min. The resulting supernatant was vacuum-dried and analyzed by mass spectrometry. In preparation, the supernatant was mixed with 100 μL of an acetonitrile/water solution for mass spectrometry analysis. The sample was mixed by vortexing and was centrifuged at 14,000× *g* and 4 °C for 15 min. The supernatant was used for sample analysis. An equal volume of the mixed solution was taken from the samples for testing as a quality control. Samples were separated using an Agilent 1290 Infinity LC ultra-high-performance liquid chromatography system with a HILIC column; the samples were stored in an automatic sampler at 4 °C. The separated samples were analyzed using a mass spectrometer (AB Triple TOF 6600). Raw data were cleaned and quality-checked using the FastQC 0.12.0 software. Principal component analysis was performed using the R package gmodels (v2.18.1) (https://CRAN.R-project.org/package=gmodels) for dimensionality reduction and quality control. The metabolites were annotated using public databases (MassBank, Metlin, and MoNA). OPLS-DA and univariate t-tests were combined to screen DEMs. Screening criteria included VIP ≥ 1 from OPLS-DA models and *p* < 0.05 from T-tests. The KEGG database was used for functional enrichment analysis and annotation of the identified metabolites.

#### 5.3.3. Determination of the Downstream Substances of Proteins

The fresh root systems of *P. crinitum* were subjected to various levels of Pb stress. The roots (0.05 g) were crushed to a powdery consistency in liquid nitrogen using a mortar and pestle, and the powder that was produced was added to a 1.5-mL centrifuge tube. Phosphate-buffered saline (0.45 mL) was added to prepare a 10% tissue homogenate. The homogenate was thoroughly homogenized using a vortex mixer and centrifuged in a chilled high-speed centrifuge for 15 min at 3000 rpm. The resulting supernatant was collected for the determination of the downstream protein species.

The amount of GSH was determined using the 5,5′-dithiobis-(2-nitrobenzoic acid) test [75]. Maltodextrin was extracted using a maltodextrin ELISA detection kit (Shanghai Enzyme and Australia Biotechnology Co. Ltd., Shanghai, China). Glutamic acid and jasmonic acid were extracted using plant glutamic acid and plant jasmonic acid ELISA detection kits (Jeremy Biotechnology Co., Ltd., Shanghai, China), respectively. Subsequently, the protein content was measured using a multi-mode microplate reader (SpectraMax iD5).

#### 5.3.4. RNA Extraction and RT-qPCR

The RNAprep Pure Plant Plus Kit was used to extract total RNA from the root systems (Tiangen Biotech Co., Ltd., Beijing, China). The concentration and quality of the extracted total RNA were assessed using a Denovix micro-spectrophotometer. cDNA was obtained by reverse transcription using the Uni All-in-one First-Strand cDNA Synthesis Super Mix for qPCR reverse transcription kit produced by Quanshikjin Company (Beijing, China). The reaction system was as follows: 5 μL of template RNA, 4 μL of 5x TransScript® All-in-One SuperMix for PCR, 1 μL of gDNA Remover, and 10 μL of RNase-Free Water. The reaction procedure: Incubate at 50 °C for 5 min, then inactivate TransScript^®^ Uni RT/RI and gRNA Remover by heating at 85 °C for 5 s. The obtained cDNA stock solution was diluted 5-fold for use in subsequent experiments [76]. 18S rRNA was selected as the reference gene [76] and candidate gene-specific primers were designed using the Primer-BLAST tool of the NCBI database and validated with DNAMAN 8 software. Synthesized by Fuzhou Shangya Biotechnology (Fuzhou, China). Detailed information on the sequences of the candidate gene-specific primers is provided in Supplementary Table S1. Candidate gene expression was measured using the HRbio™ qPCR SYBR Green Master Mix reagent (Fujian Herui Biotechnology Co., Ltd., Fuzhou, China) and QuantStudio 1 PLUS PCR. The reaction system consists of: 0.4 μL each of forward and reverse primers (10 μM), 1 μL cDNA, 10 μL HRbio™ qPCR SYBR Green Master Mix (Low Rox Plus), and 8.2 μL nuclease-free water, for a total volume of 20 μL. Amplification was performed using a two-step protocol: initial denaturation at 95 °C for 5 min, followed by 40 cycles of denaturation at 95 °C for 10 s and combined annealing/extension at 60 °C for 30 s. The relative expression levels of the candidate genes were ascertained using the 2^−ΔΔCT^ technique [77]. Each sample was set up with three biological replicates, and each biological replicate contained two technical replicates.

#### 5.3.5. PRM Measurement

Protein extraction and enzymatic digestion were performed using a previously described label-free proteomic approach and the acquired data were used to construct a spectral library. After desalting and freeze-drying, the peptides were dissolved in solvent A (0.1% formic acid in water) once more and subjected to liquid chromatography-tandem mass spectrometry analysis, after which 400 ng of each sample was added to an AUR3-15075C18 analytical column, and sample separation was performed on a 30-min gradient. The column temperature was maintained at 50 °C and the column flow rate was maintained at 300 nL/min. Finally, the column was equilibrated using 4% solvent B for 3 min. The mass spectrometer was set to operate in PRM parallel accumulation–serial fragmentation mode for analysis.

### 5.4. Statistical Analyses

Principal component analysis was performed using R (http://www.r-project.org/) (accessed on 2 June 2025). The R package pheatmap (v1.0.12) was used for clustering analysis of differential proteins and metabolites and for plotting heatmaps. OPLS-DA was performed using the ropls package to complete the task in R. OmicShare (https://www.omicshare.com) (accessed on 18 June 2025)was used for KEGG enrichment analysis and heatmap plotting. Protein–metabolite network diagrams were created using the Cytoscape 3.7 software. All statistical analyses were performed using SPSS version 22 (IBM Corp, Armonk, NY, USA). One-way analysis of variance and Duncan’s multiple comparison test were performed to test for significance (*p* < 0.05). Prism 9.5 (GraphPad, San Diego, CA, USA) was used to create box and bar graphs showing the relative expression of proteins that respond to Pb stress and their downstream metabolites. The metabolic circuit diagrams were drawn using Adobe Illustrator 2023 (Adobe, San Jose, CA, USA).

## Data Availability

The data reported in this paper have been deposited in the OMIX, China National Center for Bioinformation/Beijing Institute of Genomics, Chinese Academy of Sciences (https://ngdc.cncb.ac.cn/omix (accessed on 1 September 2025): Proteomics data accession no. OMIX011692, Metabolomics data accession no. OMIX011693).

## Supplementary Materials

The following supporting information can be downloaded at https://www.mdpi.com/article/10.3390/plants15152288/s1: Table S1: Candidate Pb-resistant gene primer sequences; Table S2. Protein Identification Summary Table; Table S3: PRM analysis of 6 Pb-tolerant proteins; Table S4. Comparative analysis of differentially expressed proteins based on label-free and PRM methods.; Figure S1: Chromatogram of secondary fragment ions extracted from the corresponding precursor ion of peptide segment. Note: In the chromatogram, the x-axis represents the retention time at which the peptide (precursor ion) and its fragment ions are eluted by the liquid chromatography mobile phase, flow out of the column, and are captured by the mass spectrometer detector. The y-axis reflects the signal intensity of the target fragment ions detected by the mass spectrometer at a specific retention time. The peak area (i.e., the region enclosed by the curve and the baseline, typically highlighted by gray shading) is proportional to the actual abundance/concentration of the peptide in the sample. GCK represents the control group of P. crinitum roots, and GPb400, GPb800, and GPb1000 represent the lead-treated groups of P. crinitum roots.; Figure S2. Results of RNA Agarose Gel Electrophoresis.
