# Supplementary material for: Glutathione and Jasmonic Acid Biosynthesis Coordinate Antioxidant and Hormonal Responses to Alleviate Lead Toxicity in Pogonatherum crinitum Roots
Source: Plants (Basel). 2026 Jul 26;15(15):2288. doi: 10.3390/plants15152288 (PMC13468550; doi:10.3390/plants15152288)

**Supplemental Table:**

**Table S1.** Candidate Pb-resistant gene primer sequences.

| GeneID | Primer sequences | Tm | GC% |
|---|---|---|---|
| BGLU30 | F: TGGTCCAGGCAATGGAGAAC | 59.96 | 55.00 |
| | R: TTGAACAACGATACCGGCGA | 60.04 | 50.00 |
| GST4 | F: CCATGTGTAGTAGCGGCCAT | 59.89 | 55.0 |
| | R: GTGGTGCCTCGATCTGTAGT | 59.18 | 55.0 |
| GSTU25 | F: AAAGAACGGCTTGTCTCCGA | 59.61 | 50.00 |
| | R: CCAGGTTCTGGGCAGACTTC | 60.32 | 60.00 |
| GSTU1 | F: CGAGCGGTTTGGGGAGATTA | 59.82 | 55 |
| | R: CCGAGCCTCTTCTTCATCCC | 59.89 | 60 |
| GRC2 | F: GGACCCAATCTATGGCGAGG | 59.96 | 60.00 |
| | R: AGCTGATGGGGTGAAATGGG | 60.03 | 55.00 |
| ACX4 | F: ACAATTCTCAAGCAGCCGTG | 59.12 | 50.0 |
| | R: GTTCCGTGCCCAGACAAGAG | 60.95 | 60.0 |
| OPR1 | F: AGCTGTTCCACCGTGTTGT | 59.78 | 52.63 |
| | R: TTTGGCAGATGAAGAAGGCG | 58.83 | 50.0 |

| | | | |
|---|---|---|---|
| BMY1 | F: CATGTCATGTCACCAGTGCG | 59.55 | 55 |
| | R: GTCCACCCCAATCGTCAGG | 60.08 | 63.16 |
| GSTU6 | F: AAGACGACCTCAAGCTGCTC | 60.04 | 55 |
| | R: GGCAGGAAGGATTGAAGGCT | 60.03 | 55 |

**Table S2.** Protein Identification Summary Table

| Title | Number |
|---|---|
| Total spectrums | 572845 |
| Matched spectrums | 213749 |
| Peptides | 35012 |
| Unique peptides | 30828 |
| Identified proteins | 7773 |
| Quantifiable proteins | 5328 |

**Table S3.** PRM analysis of 6 Pb-tolerant proteins.

| Protein | CK | Pb400 | Pb800 | Pb1000 | Pb400/CK | Pb800/ CK | Pb1000/ CK |
|---|---|---|---|---|---|---|---|
| BGLU30 | 6.58±0.52b | 7.64±0.75ab | 8.20±0.70a | 8.93±0.79a | 1.16 ↑ | 1.24 ↑ | 1.35 ↑ |

| | | | | | | | |
|---|---|---|---|---|---|---|---|
| BMY1 | 3.83±0.03a | 4.24±0.70a | 4.28±0.10a | 4.72±1.31a | 1.10 ↑ | 1.11 ↑ | 1.23 ↑ |
| GST4 | 6.72±2.06b | 8.92±0.03ab | 8.44±0.16ab | 9.76±1.60a | 1.32 ↑ | 1.25 ↑ | 1.45 ↑ |
| GSTU1 | 9.31±0.90a | 10.05±0.60a | 9.74±0.65a | 10.73±0.78a | 1.08 ↑ | 1.04 ↑ | 1.15 ↑ |
| GRC2 | 6.58±1.10b | 7.65±0.52ab | 8.18±0.36a | 8.93±0.71a | 1.16 ↑ | 1.24 ↑ | 1.35 ↑ |
| ACX4 | 7.42±1.21b | 8.68±1.93ab | 8.37±0.64ab | 8.91±0.10a | 1.16 ↑ | 1.12 ↑ | 1.20 ↑ |

**Table S4. Comparative analysis of differentially expressed proteins based on Lable free and PRM methods.**

| Protein Name | Lable free CK | Lable free Pb1000 | Fold Change Lable free | PRM CK | PRM Pb1000 | Fold Change PRM | Consistency of Lable free and PRM |
|---|---|---|---|---|---|---|---|
| BGLU30 | 0.78 | 1.06 | 1.35 | 6.58 | 8.93 | 1.35 | Yes |
| BMY1 | 0.75 | 1.26 | 1.68 | 3.83 | 4.72 | 1.23 | Yes |
| GST4 | 0.49 | 1.55 | 3.16 | 6.72 | 9.76 | 1.45 | Yes |
| GSTU1 | 0.60 | 1.46 | 2.43 | 9.31 | 10.73 | 1.15 | Yes |
| GRC2 | 0.73 | 1.31 | 1.79 | 6.58 | 8.93 | 1.35 | Yes |
| ACX4 | 0.78 | 1.25 | 2.38 | 7.42 | 8.91 | 1.20 | Yes |

## Supplemental Figure:

**Figure S1.** Chromatogram of secondary fragment ions extracted from the corresponding precursor ion of peptide segment. Note: In the chromatogram, the x-axis represents the retention time at which the peptide (precursor ion) and its fragment ions are eluted by the liquid chromatography mobile phase, flow out of the column, and are captured by the mass spectrometer detector. The y-axis reflects the signal intensity of the target fragment ions detected by the mass spectrometer at a specific retention time. The peak area (i.e., the region enclosed by the curve and the baseline, typically highlighted by gray shading) is proportional to the actual abundance/concentration of the peptide in the sample. GCK represents the control group of *P. crinitum* roots, and GPb400, GPb800, and GPb1000 represent the lead-treated groups of *P. crinitum* roots.

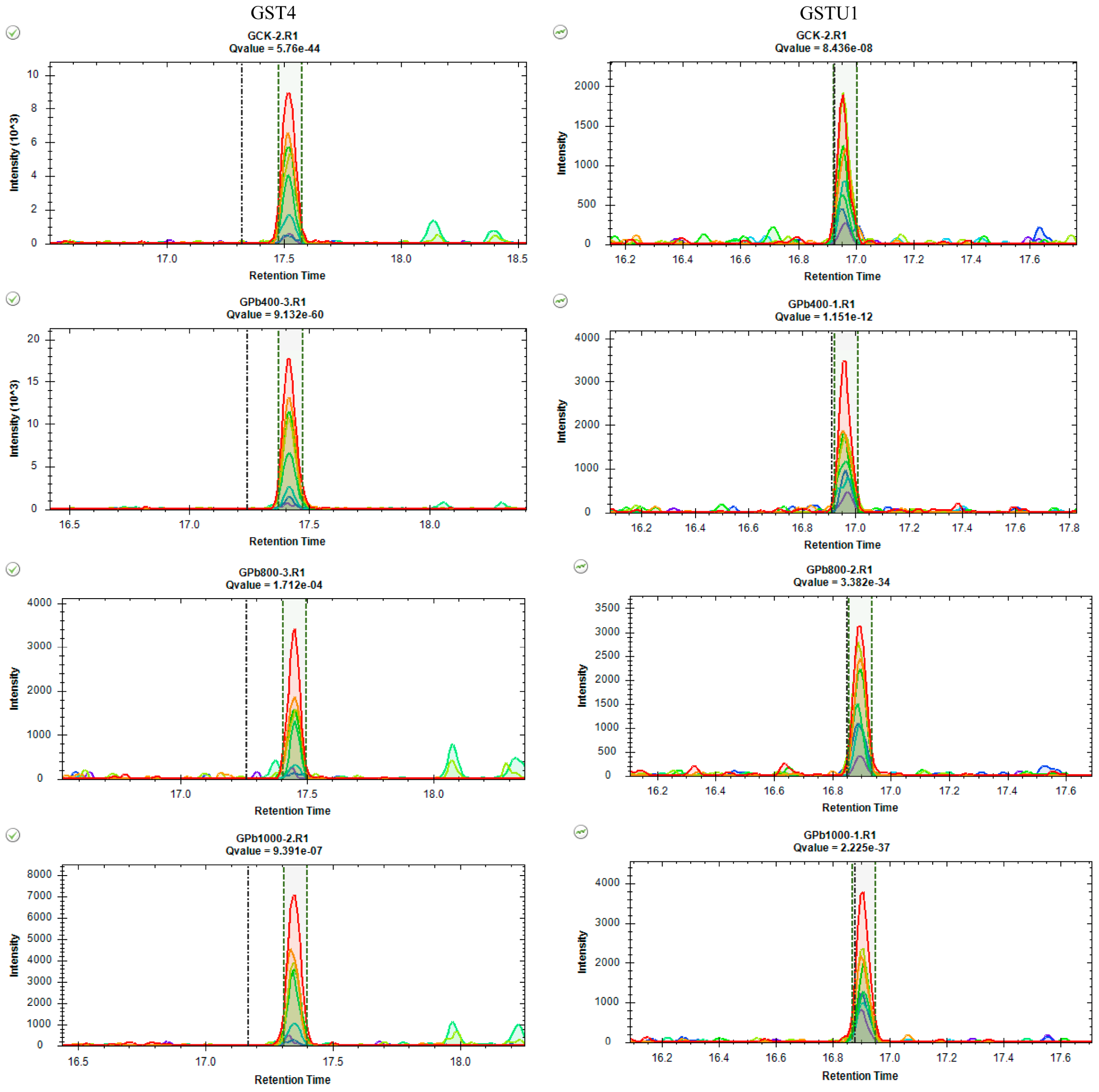

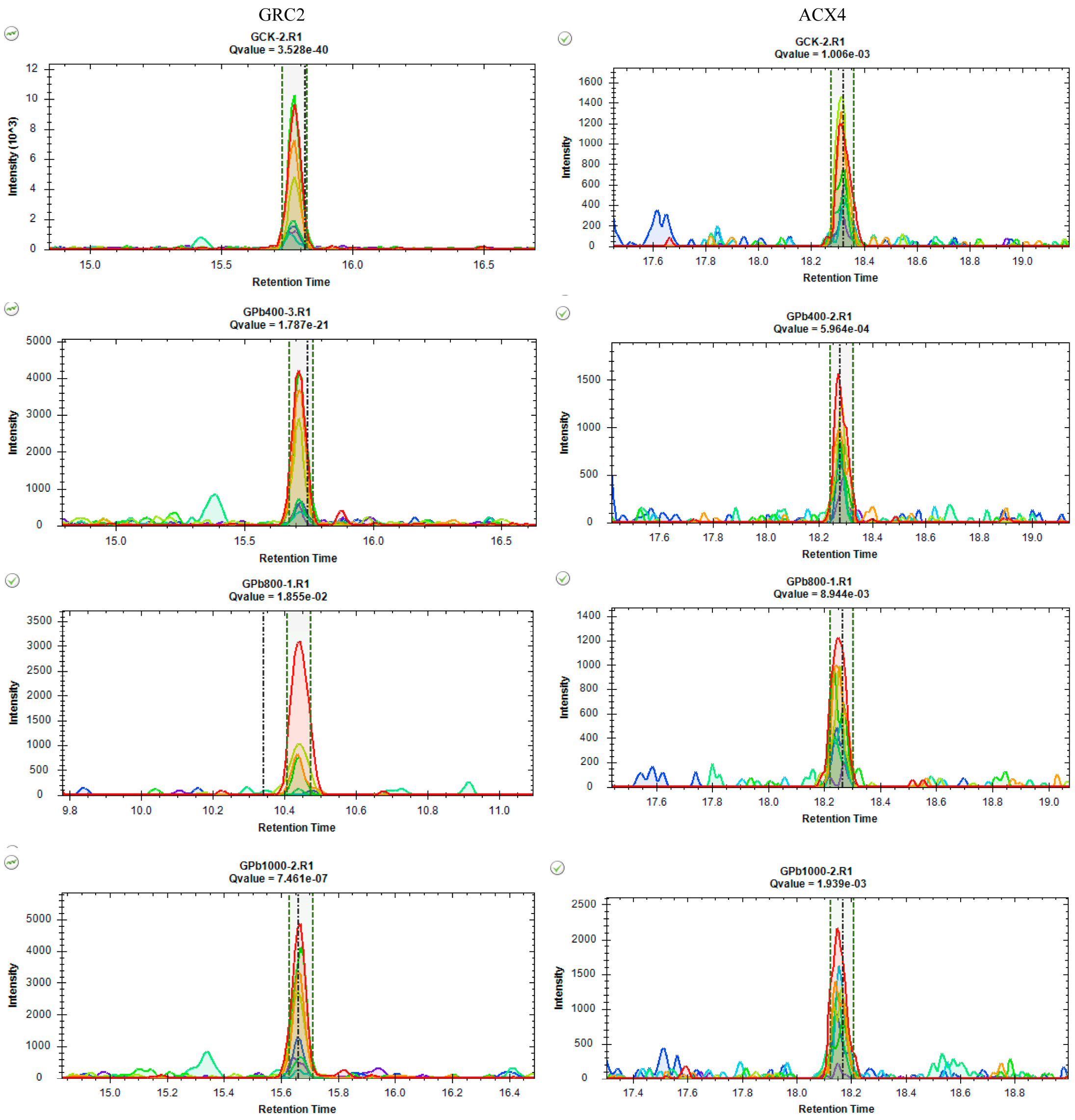
GRC2
ACX4
GCK-2.R1
Qvalue = 3.528e-40
Intensity (10^3)
Retention Time
GCK-2.R1
Qvalue = 1.006e-03
Intensity
Retention Time
GPb400-3.R1
Qvalue = 1.787e-21
Intensity
Retention Time
GPb400-2.R1
Qvalue = 5.964e-04
Intensity
Retention Time
GPb800-1.R1
Qvalue = 1.855e-02
Intensity
Retention Time
GPb800-1.R1
Qvalue = 8.944e-03
Intensity
Retention Time
GPb1000-2.R1
Qvalue = 7.461e-07
Intensity
Retention Time
GPb1000-2.R1
Qvalue = 1.939e-03
Intensity
Retention Time

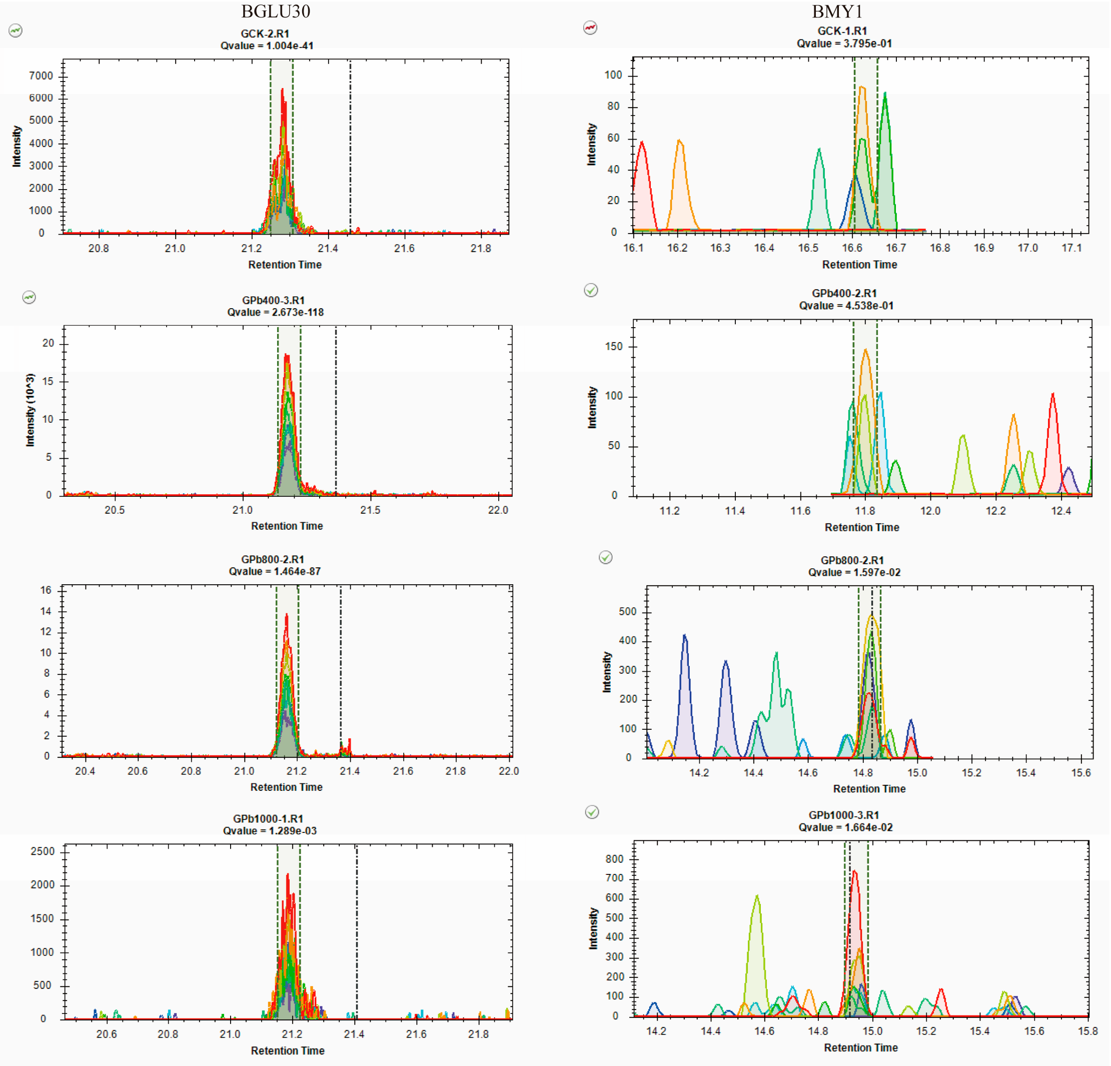


**Figure S2.** Results of RNA Agarose Gel Electrophoresis.

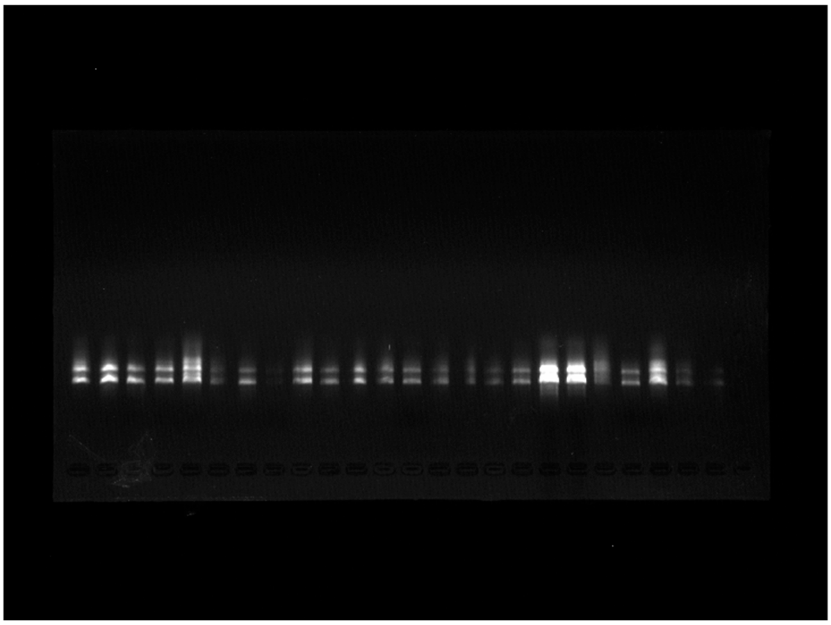

Supplement: Supplementary file 1 [file plants-15-02288-s001.zip › plants-4412892-supplementary.pdf]
